# Adenosine-generating ovarian cancer cells attract myeloid cells which differentiate into adenosine-generating tumor associated macrophages – a self-amplifying, CD39- and CD73-dependent mechanism for tumor immune escape

**DOI:** 10.1186/s40425-016-0154-9

**Published:** 2016-08-16

**Authors:** Itsaso Montalbán del Barrio, Cornelia Penski, Laura Schlahsa, Roland G. Stein, Joachim Diessner, Achim Wöckel, Johannes Dietl, Manfred B. Lutz, Michel Mittelbronn, Jörg Wischhusen, Sebastian F. M. Häusler

**Affiliations:** 1 Department of Obstetrics and Gynaecology, University of Würzburg, School of Medicine, Josef-Schneider-Strasse 4, 97080 Würzburg, Germany; 2 Interdisciplinary Centre for Clinical Research, University of Würzburg, School of Medicine, Würzburg, Germany; 3 German Cancer Consortium (DKTK), German Cancer Research Center (DKFZ), Heidelberg, Germany; 4 Edinger Institute (Neurological Institute), Goethe University, Frankfurt, Germany; 5 Institute of Virology and Immunobiology, University of Würzburg, Versbacherstrasse 7, 97078 Würzburg, Germany

**Keywords:** Ovarian cancer, Adenosine, CD39, CD73, Tumor associated macrophages, Immune escape

## Abstract

**Background:**

Ovarian cancer (OvCA) tissues show abundant expression of the ectonucleotidases CD39 and CD73 which generate immunomodulatory adenosine, thereby inhibiting cytotoxic lymphocytes. Little, however, is known about the effect of adenosine on myeloid cells. Considering that tumor associated macrophages (TAM) and myeloid-derived suppressor cells (MDSC) constitute up to 20 % of OvCA tissue, we investigated the effect of adenosine on myeloid cells and explored a possible contribution of myeloid cells to adenosine generation in vitro and ex vivo.

**Methods:**

Monocytes were used as human blood-derived myeloid cells. After co-incubation with SK-OV-3 or OAW-42 OvCA cells, monocyte migration was determined in transwell assays. For conversion into M2-polarized “TAM-like” macrophages, monocytes were co-incubated with OAW-42 cells. Ex vivo TAMs were obtained from OvCA ascites. Macrophage phenotypes were investigated by intracellular staining for IL-10 and IL-12. CD39 and CD73 expression were assessed by FACS analysis both on in vitro-induced TAM-like macrophages and on ascites-derived ex situ-TAMs. Myeloid cells in solid tumor tissue were analyzed by immunohistochemistry. Generation of biologically active adenosine by TAM-like macrophages was measured in luciferase-based reporter assays. Functional effects of adenosine were investigated in proliferation-experiments with CD4^+^ T cells and specific inhibitors.

**Results:**

When CD39 or CD73 activity on OvCA cells were blocked, the migration of monocytes towards OvCA cells was significantly decreased. In vivo, myeloid cells in solid ovarian cancer tissue were found to express CD39 whereas CD73 was mainly detected on stromal fibroblasts. Ex situ-TAMs and in vitro differentiated TAM-like cells, however, upregulated the expression of CD39 and CD73 compared to monocytes or M1 macrophages. Expression of ectonucleotidases also translated into increased levels of biologically active adenosine. Accordingly, co-incubation with these TAMs suppressed CD4^+^ T cell proliferation which could be rescued via blockade of CD39 or CD73.

**Conclusion:**

Adenosine generated by OvCA cells likely contributes to the recruitment of TAMs which further amplify adenosine-dependent immunosuppression via additional ectonucleotidase activity. In solid ovarian cancer tissue, TAMs express CD39 while CD73 is found on stromal fibroblasts. Accordingly, small molecule inhibitors of CD39 or CD73 could improve immune responses in ovarian cancer.

**Electronic supplementary material:**

The online version of this article (doi:10.1186/s40425-016-0154-9) contains supplementary material, which is available to authorized users.

## Background

Immune function in the tumor microenvironment is shaped by tissue-specific and tumor-derived signals [1] which often decrease the effectiveness of anti-tumor immune responses. This is particularly relevant for malignancies like ovarian cancer (OvCA) where immunological processes like infiltration with cytotoxic [2] or regulatory T cells (T_reg_) [3] heavily affect the prognosis [1, 4, 5]. In this context, we and others have identified tumor-derived adenosine as important immunomodulatory factor [6–10]. Adenosine which signals via four specific receptors [11, 12] can activate several cellular responses which mainly serve to protect the tissue from excessive inflammatory responses [12–16]. Sustained high levels of adenosine can, however, turn harmful by triggering immune suppression or by activating unremitting wound-healing [17, 18]. Adenosine levels are thus kept low under physiological conditions, but increased by stimuli such as inflammation, trauma, hypoxia or ischemia [12]. In ovarian cancer, high levels of adenosine are due to tumor-specific expression of the ectonucleotidases CD39/ENTPD1 and CD73/ecto-5′-nucleotidase [7] which synergistically catalyze the degradation of extracellular immune-stimulatory ATP to immune-inhibitory adenosine. First, ATP or ADP are hydrolysed by CD39 to AMP which in turn is dephosphorylated to adenosine by CD73 [19–22]. Adenosine can then suppress anti-tumoral functions of CD4^+^ or CD8^+^ T cells and Natural Killer (NK) cells [7, 9]. This mechanism of immunosuppression was also proposed for CD4^+^CD25^+^FoxP3^+^ T_reg_ cells [20]. OvCA cells, however, seem to greatly outperform T_reg_ regarding adenosine generation [7].

Myeloid-derived suppressor cells (M-MDSC) and tumor associated macrophages (TAM) are infiltrating myeloid cells which, similar to T_reg_, significantly impact survival as they enhance progression and metastasis [23–26]. In ovarian cancer they build up to 20 % of the tumor volume [27]. Monocytic and polymorphonuclear MDSC are recruited by tumors where they can remain in their relatively immature state. The tumor microenvironment, however, promotes further differentiation of monocytic MDSC into TAM [28, 29]. TAM are alternatively activated M2-macrophages which (as opposed to inflammatory M1-macrophages) orchestrate anti-inflammatory, mostly immunosuppressive reactions [25, 30]. By producing reactive oxygen species (ROS), nitric oxide (NO), indoleamine-2,3-dioxygenase (IDO) and by secreting high levels of immunosuppressive cytokines like IL-10 and TGF-β [27], MDSC and TAM significantly shape the immune contexture of ovarian and other solid tumors. Clinically, abundance of MDSC and TAM has been linked to poor outcome in various malignancies [31, 32] including ovarian cancers of the serous and mucinous subtype [33, 34]. Interestingly, myeloid cells seem to accumulate in hypoxic tissue areas [27, 31] where low oxygen pressure leads to necrotic cell death with concomitant ATP release. CD39 and CD73 can, however, convert this pro-inflammatory signal [35–38] into immunosuppressive adenosine.

While the suppressive effect of adenosine on several immune cells such as CD4^+^ and CD8^+^ T cells and Natural Killer (NK) cells is well documented, little is known about the effect of tumor-derived adenosine on myeloid cells. Moreover, the available data are all based on murine cells [39–41].

These findings prompted us to investigate the effect of OvCA-derived adenosine on human monocytes which are the circulating precursors of both monocytic MDSC and TAM and can be obtained from peripheral blood. In addition, we explored a possible regulation of CD39 and CD73 expression on macrophages in response to the respective mode of differentiation. Finally, we also performed proliferation assays in vitro in order to functionally confirm an immunomodulatory role of CD39 and CD73 on M2-macrophages (or TAM). Within the limits of an ex vivo study confined to the use of PBMC-derived monocytes to approximate MDC and TAM, we have thereby obtained data that suggest a role for adenosine in the accumulation and function of tumor-associated macrophages.

## Methods

### Bioinformatic analysis

Gene expression data were obtained from from 285 ovarian cancer samples, from the AOCS (Australian Ovarian Cancer Study) (GEO ID: GSE9891). Gene expression was profiled on the affymetrix U133_plus2 platform. Expression data are publicly available from the R2 bioinformatics program (http://R2.amc.nl). Bioinformatic analysis of microarray data was conducted with the R2 tool by first searching for genes correlated with CD39 in ovarian cancer. Subsequently, pairwise correlation analyses were performed as indicated. To correlate expression of CD39 and CD73 with overall survival inovarian cancer patients, we have used the the free online tool Kaplan-Meier plotter (http://kmplot.com/analysis/index.php?p=service&cancer=ovar) [42]. 347 patients with stage 3 and 4 (high-grade) serous ovarian cancer were included, regardless of p53 status, CA125 levels, optimal/suboptimal debulking or chemotherapy. A combined classifier comprising gene expression data for both CD39/ ENTPD1 (Affymetrix ID 228585) and CD73/NT5E (Affymetrix ID 203939) was bioinformatically computed and survival probabilities were calculated according to Kaplan-Meier statistics including 95 % confidence intervals (CIs). For comparison, two-sided log-rank tests were used.

### Immunohistochemistry and immunofluorescence

All tissue specimens (10 ovarian cancer, one healthy ovary) were provided by the University Hospital Würzburg (Würzburg, Germany). Samples had been evaluated by at least two pathologists in routine diagnostics as serous-papillary OvCA or benign ovary. All human tissue specimens were cut with a microtome (3 μm thickness) and placed on SuperFrost-Plus slides (Microm International, Walldorf, Germany). IgG2a-mouse-anti-human CD39 antibody (dilution 1:10; Biozol, Eching, Germany) and IgG-rabbit-anti-human CD73 antibody (dilution 1:500; Sigma-Aldrich, St. Louis, MO, USA) were used for immunohistochemistry. Tissue labelling was performed using the DiscoveryXT immunohistochemistry system (Ventana/Roche, Strasbourg, France). After a cell conditioning pre-treatment a 4 min blocking step was performed. The primary antibodies were applied for 32 min, followed by a secondary OmniMap anti-mouse HRP (horseradish peroxidase) (Ventana) for 16 min incubation for anti-CD39 staining and a universal secondary HRP antibody (Ventana) for 32 min for anti-CD73 staining. For diaminobenzidine (DAB) visualization, the sections were incubated with one drop of DAB CM and one drop of H_2_O_2_ CM (Ventana) for 8 min, followed by incubation with a copper enhancer (Ventana) for 4 min. Human tonsil (for anti-CD39) and human placenta (for anti-CD73) were used as positive controls. Negative controls were performed by omission of the first antibody. Finally, all sections were then washed, counterstained with hematoxylin and mounted.

For immunofluorescent stainings, 3 μm thick slides were deparaffinized with xylene and rehydrated in a descending alcohol sequence. Heat pre-treatment for antigen retrieval was performed with citrate buffer (pH 6.0) for 40 min, followed by a blocking step for 30 min (Roti-Immuno Block, dilution 1:10; Roth, Karlsruhe, Germany) at room temperature. Sections were incubated with primary antibodies for 1 h at room temperature (anti-CD39: dilution 1:10 and anti-CD73: dilution 1:500) and subsequently labelled with secondary antibodies for 1 h each (for CD39: Alexa Fluor488, dilution 1:250, donkey anti-mouse IgG, Invitrogen, Darmstadt, Germany; for anti-CD73: Alexa Fluor488, dilution 1:250, goat anti-rabbit IgG, Invitrogen). Next, the primary antibodies for the double staining were added for 1 h at room temperature (for anti-CD39: rabbit-anti-human-Iba-1 (dilution 1:1000; Wako, Richmond, VA, USA; for anti-CD73: mouse-anti-human-CD68 (dilution 1:500; Dako, Hamburg, Germany), followed by an additional secondary antibody for 1 h (for anti-Iba1: Alexa Fluor568, dilution 1:250, donkey anti-rabbit IgG, Invitrogen; for anti-CD68: Alexa Fluor568, dilution 1:250, goat anti-mouse IgG, Invitrogen). Nuclear counterstaining was performed using Topro-3 (dilution: 1:1000; Invitrogen) followed by sudan black for 5 min to block autofluorescence. Fluorescence images were analyzed and recorded on a Nikon C1si (Nikon, Japan) confocal microscope, using the EZ-C1 software. After recording, digital images were further processed and adjusted for brightness, contrast and color balance.

### Cell culture

The human ovarian cancer cell lines SK-OV-3 or OAW-42 were cultured in RPMI 1640 medium with 10 % FCS, 0.02 % sodium pyruvate, penicillin (100 IU/ml) and streptomycin (100 μg/ml) (all from Gibco, Karlsruhe, Germany). Cell line identity was confirmed via single tandem repeat fingerprinting by the Deutsche Sammlung für Mikroorganismen und Zellkulturen (Braunschweig, Germany). Tumor associated macrophages were isolated from ascites from OvCA patients (*n* = 9) using anti-CD14 coated magnetic beads (Miltenyi Biotec, Bergisch Gladbach, Germany) according to the manufacturer’s recommendations. For control purposes peritoneal macrophages were isolated from patients with benign ascites (*n* = 8). All ascitic fluid punctures were performed for medical needs.

### Migration assay

Peripheral blood mononuclear cells (PBMC) were isolated from the blood of healthy volunteers. To enrich for monocytes, a two-step gradient centrifugation protocol was used, starting with a standard Ficoll-based separation medium (Biocoll, Biochrom, Berlin, Germany) followed by a Percoll gradient (Easycoll separation medium, Biochrom, Berlin, Germany). 500.000 monocytes per well were then placed in the upper inserts of 24-well Transwell plates (pore diameter 8 μm, membrane thickness 10 μm, cell growth area 0.33 cm^2^, Corning, Tewksbury, USA) while 200.000 OAW-42 or SK-OV-3 cells were placed in the corresponding compartments at the bottom of the plate. All assays were conducted in RPMI 1640 medium with 5 % human AB serum (PAA) (500 μl per well). After an incubation period of 6 h, all cells from the bottom plates were analyzed by flow cytometry. Monocytes were identified with anti CD11c-FITC (Immunotools, Friesoythe, Germany) whereas OvCA cells were stained with EpCam-APC (BioLegend, San Diego, USA) (both antibodies used at 1:100 dilution). Dead cells were excluded via co-staining with 7-aminoactinomycin D. To quantify the relative migration rate, a standard curve was generated from several samples containing 2x10^5^ cancer cells together with different numbers of monocytes (0 -10^6^ cells). To block CD39 or, respectively, CD73 activity during the transwell co-culture, ARL 67156 (250 μM, Tocris, Bristol, UK), α,β-methyleneadenosine-5′-diphosphate (“APCP”, 100 μM, Sigma, Deisenhofen, Germany) or appropriate solvent controls were used (Crack et al., 1995; Krug et al., 1973). As positive control, the metabolically stable adenosine receptor agonist adenosine-5′-N-ethylcarboxamide (NECA, Tocris) was employed at 100 nM. To control for effects of adenosine on chemokinesis, a control without tumor cells but with equal amounts of NECA in both compartments was performed.

### RT^2^ Profiler PCR array for adenosine-dependent modulation of chemokines and chemokine receptors on monocytes

To investigate if NECA has an effect on the expression of chemokines or chemokine receptors in monocytes, mRNA expression on CD14^+^ bead-purified monocytes was assessed 3h after NECA treatment in comparison to an untreated control. 280ng total RNA per sample were used for cDNA synthesis (RT^2^ First Strand Kit, Qiagen, Hilden, Germany). The RT^2^ Profiler PCR array for human chemokines & receptors and the RT^2^ SYBR Green ROX qPCR mastermix (both from Qiagen) were used according to the manufacturer’s instructions. Assays were run on a StepOnePlus RealTime-PCR cycler (thermofisher, Darmstadt, Germany). For data analysis, the same threshold was applied for each plate and data were analyzed in the Qiagen Analysis Webportal. Candidate molecules appearing to be regulated by NECA in the arrays where then validated by qRT-PCR (Additional file 1).

### Determination of M1 and M2 macrophage polarization and of CD39 and CD73 expression

Monocytes were isolated from healthy volunteers as described above and matured in Lumox dishes (Greiner bio-one, Frickenhausen, Germany) during 7 days in RPMI 1640 with 5 % human AB serum at 37 °C. Polarization towards M1-phenotype was induced with human recombinant IFN-γ [1 μg/ml] and LPS [10 μg/ml] (both from Peprotech, Hamburg, Germany) for 48h at 37 °C in RPMI 1640 medium with 5 % human AB serum. M2-phenotype macrophages were generated by co-culture of matured monocytes with OAW-42 cells in transwell plates (pore size 1 μm) for 48h at 37 °C in RPMI 1640 with 5 % human AB serum [43]. M1- and M2-polarization were confirmed by flow cytometric analysis of intracellular IL-10- and IL-12-levels [44]; to this aim the cells were stained with anti-IL10-FITC (Miltenyi) and anti-IL12-APC (Biolegend) at 1:100 dilution. Based on the expression of CD68, macrophage purity was found to be >90 %. In addition, arginase activity was determined in a colorimetric assay based on urea production. Polarized macrophages were washed twice with ice-cold PBS, scraped and centrifuged at 1,200 rpm for 5 min. Cell pellets were resuspended into 200 μl lysis buffer (50 mM Tris–HCl (pH 7.5), 0.1 mM EDTA, 0.1 mM ethylene glycol-bis(β-aminoethyl ether)-*N*, *N*, *N*′, *N*′-tetraacetic acid, 1 mM dithiothreitol, 1 μg/ml leupeptin, 1 μg/ml aprotinin, and 0.1 mM phenylmethylsulfonyl fluoride). Cell lysates (50 μl) were added into 50 μl of 50 mM Tris–HCl (pH 7.5) containing 10 mM MnCl_2_. Arginase was then activated by heating the mixture at 55–60 °C for 10 min. To allow for catalytic conversion of L-arginine by arginase, lysates were incubated with 50 μl of L-arginine (0.5 M; pH 9.7) at 37 °C for 1 h, before the reaction was stopped by addition of 400 μl acid solution mixture (1 H_2_SO_4_:3 H_3_PO_4_:7 H_2_O). For visualization, 25 μl of 9 % α-Isonitrosopropiophenone (in 100 % ethanol) were added to the mixture and heated at 100 °C for 45 min. Once the samples had been incubated in the dark for 10 min at RT, the urea concentration was determined by measuring the absorbance at 550 nm in a spectrophotometer. The rate of urea production was used as an index for arginase activity.

Ectonucleotidase expression on M1/M2-macrophages and on patient-derived TAMs was assessed with anti-CD73-PE (BioLegend) or anti-CD39-PECy7 (Miltenyi) at 1:100. Specific Fluorescence Indices (SFI) were calculated for each surface marker by dividing the signal intensity obtained with the specific by the signal intensity measured with an irrelevant isotype-matched control antibody. All flow cytometry analyses were performed using a FACSCalibur flow cytometer (BD Biosciences, San Jose, USA).

CD39 and CD73 mRNA transcript levels were quantified from cDNA by semi-quantitative real time PCR (qRT-PCR) using the ABsolute Blue QPCR SYBR Green low Rox mix kit and the following primer pairs: CGGCTACCACATCCAAGGAA (frw) and GCTGGAATTACCGCGGCT (rev) for 18S; GTAAGTGACCTTTACAAGACCC (frw) and TGCTGGAATGGAAGAGTCATC (rev) for CD39; GGCTCCTCTCAATCATGCCG (frw) and CAGAACATTTCATCCGTGTGT (rev) for CD73. Purity of the PCR products was assessed based on the dissociation curve. All samples were measured in duplicate and Ct (cycle threshold)-values were within ≤0.5 cycles. mRNA expression was quantified relative to the expression 18S RNA which was used as control.

### Adenosine production via CD39 and CD73

Measurement of biologically active adenosine was performed as described before [7, 45]. Briefly, ADORA2A-overexpressing HEK-293 cells were transiently transfected with the luciferase-encoding RIP1-CRE.luc^+^ cAMP-reporter plasmid [46]. Transfection efficiency was normalized via co-transfection of pRL-CMV (Promega, Madison, WI, USA). As adenosine binding to ADORA2A activates the adenylate cyclase, a corresponding firefly luciferase signal is obtained. Accordingly, a standard curve can be generated with a dynamic range from 20 nM to 40 μM adenosine. Specificity of the signal is controlled by use of HEK-293 cells without ADORA2A overexpression and by addition of the ADORA2A-specific inhibitor SCH58261 (Tocris, Bristol UK). M1- or M2-macrophages generated as described above were co-incubated at a 1:1 ratio with of RIP1-CRE.luc- and pRL-CMV-transfected HEK-293 ADORA2A^+^ cells for 4 h. Cells were lysed with passive lysis buffer (Promega) and biophotonic signals were measured using a non-commercial dual luciferase assay (Dyer et al., 2000). Adenosine concentrations were calculated based on the co-determined standard curve. All measurements were performed in triplicates using an Orion II Microplate Luminometer (Berthold Detection Systems, Pforzheim, Germany). The specific inhibitors ARL67156 for CD39 (100 μM) and APCP for CD73 (100 μM) were added to confirm that the effects depend specifically on the respective ectonucleotidases.

### Proliferation of CD4^+^ T cells in co-culture with adenosine-generating cells

CD4 T cell proliferation was measured as before [7]. CD4^+^ cells were isolated from PBMC using the CD4^+^ T cell isolation kit II (Miltenyi). Cells were then stained with 2.5 μM 5-(and-6)-carboxyfluorescein diacetate succinimidyl ester (CFSE, Invitrogen, Karlsruhe, Germany). An agonistic anti-human CD3 antibody (clone UCHT-1, ImmunoTools) was immobilized on 96 well Maxisorp-plates (Nunc, Roskilde, Denmark) by overnight-incubation in PBS (antibody concentration: 1 μg/ml). In coated wells, T cell stimulation could be induced by addition of soluble anti-human CD28 (clone 15E8, 1 μg/ml, Immunotools). In each well, 2x10^6^ T cells were co-incubated with 5x10^5^ M2 macrophages, in the absence or presence of the specific inhibitors ARL67156 for CD39 (100 μM), APCP (100 μM) for CD73 or solvent controls. As positive control, the metabolically stable adenosine receptor agonist adenosine-5′-N-ethylcarboxamide (NECA, Tocris) was used at 10 μM. T cell proliferation was determined on a FACSCalibur flow cytometer (BD Biosciences) on day 7 (all [7]).

### Statistics

For all experiments, significance was determined by Student’s *t*-test. *p*-values < 0.05 were considered to be significant. In R2 analysis, p-values were corrected for multiple testing. In flow cytometric assays at least 50,000 events were counted; two samples were considered to be significantly different (*) when they were separated by at least twice the sum of the standard deviations for the respective regions. A difference exceeding four times the sum of the respective standard deviations was considered as highly significant (**).

## Results

### Expression of CD39 and CD73 in ovarian cancer tissue is associated with poor survival and correlates with transcripts expressed in myeloid cells

**Fig. 1:**
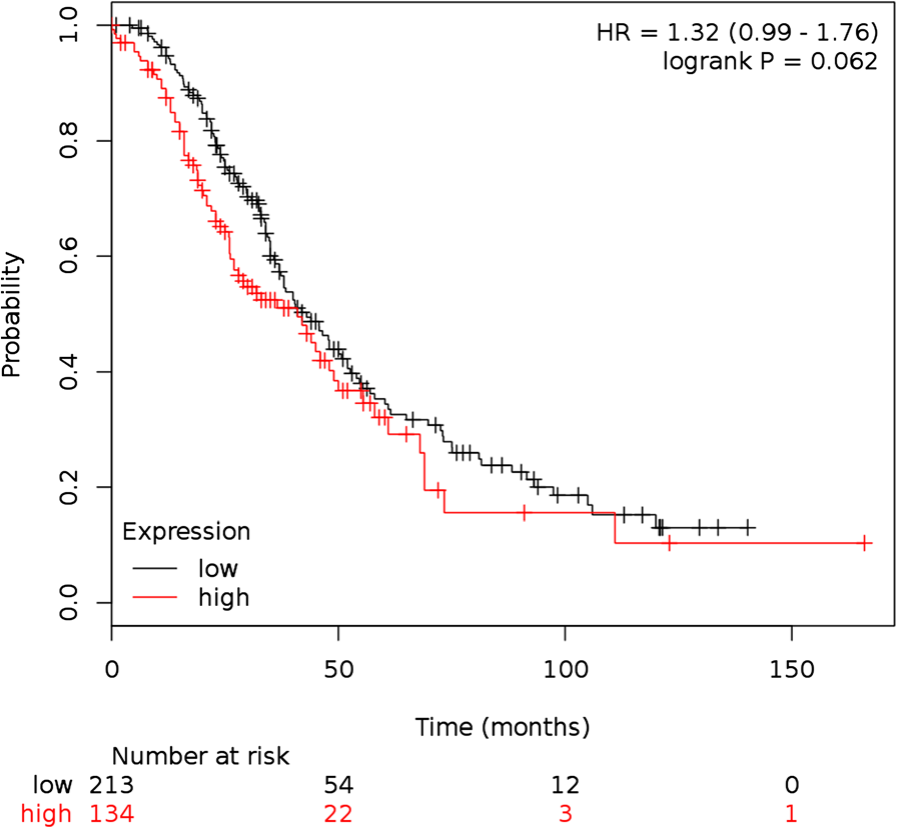
Prognostic value of CD39 and CD73 expression in patients with high-grade serous OvCA. Using the ovarian cancer dataset available via the free online tool Kaplan-Meier plotter (http://kmplot.com/analysis/index.php?p=service&cancer=ovar), expression of CD39 and CD73 was correlated with overall survival. All 347 patients with stage 3 and 4 serous OvCA were included, regardless of p53 status, CA125 levels, optimal /suboptimal debulking or chemotherapy. Based on a bioinformatically computed combined classifier comprising gene expression data for both CD39/ ENTPD1 (Affymetrix ID 228585) and CD73/NT5E (Affymetrix ID 203939), 134 patients (38.6 %) were classified as CD39/CD73^high^. Survival probabilities according to Kaplan-Meier were calculated together with 95 % confidence intervals (CIs) and compared using two-sided log-rank tests

To check for a possible correlation between CD39 and CD73 expression and survival in ovarian cancer patients, a web-based (http://kmplot.com/analysis/index.php?p=service&cancer=ovar) [42] Kaplan-Meier analysis including 347 patients with stage 3 and 4 (high-grade) serous ovarian cancer was performed. Using a combined classifier for both ectonucleotidases, CD39/CD73^high^ tumors showed a hazard ratio of 1.32 (95 % CI: 0.99 – 1.76; *p* = 0.062), thus indicating a trend towards worse survival for patients with tumors expressing CD39 and CD73 at significant levels (Fig. 1). Using the R2 database, gene expression data from 285 ovarian cancer tissues from the AOCS (Australian Ovarian Cancer Study) were screened for genes correlating with the presence of CD39 (ENTPD1(209473_at)) or, respectively, CD73 (Additional file 2: Table S1). Considering the strong correlation between both ectonucleotidases (*r* = 0.317, *p* = 4.3x10^−8^), a considerable overlap could also be expected for genes which tend to be co-expressed with either CD39 or CD73. In this context, KEGG pathway analysis using the R2 pathway finder revealed for both CD39 (*p* = 1.5x10^−5^) and CD73 (*p* = 1.6x10^−3^) a highly significant correlation with antigen processing and presentation. 42 out of 57 genes linked to this pathway show correlation with CD39 (*p* = 1.5x10^5^). Likewise, 41 out of 57 pathway-associated genes also correlated with CD73 (*p* = 1.6x10^−3^) (see Additional file 3: Table S2). Pairwise correlation analyses subsequently indicated particularly strong positive correlations with MS4A7 (membrane-spanning 4-domains subfamily A member 7) (*p* = 2.5x10^−29^), myeloid differentiation protein-2, LY96 (*p* = 2.4x10^−34^), myeloid cell nuclear differentiation antigen MNDA (*p* = 1.1x10^−29^) and colony stimulating factor 2 receptor beta, CSF2RB (*p* = 2.4x10^−33^). Correlation between CD39 and CD68 was also highly significant (*p* = 2.4x10^−10^). Further pathways linked to CD39 expression include (among others), cytokine–cytokine receptor interaction (*p* = 7.9x10^−03^), cell adhesion (*p* = 4.4x10^−05^) and leukocyte_transendothelial_migration (*p* = 8.3x10^−03^). For CD73 (NT5E), KEGG pathway analysis showed the strongest association with focal adhesion (*p* = 1.2x10^−05^), followed by cytokine_cytokine_receptor_interaction (*p* = 4.3x10^−05^), ECM_receptor_interaction (*p* = 4.7x10^−04^), antigen processing and presentation (*p* = 1.6x10^−03^) and leukocyte transendothelial migration (*p* = 1.9x10^−03^) (see Additional file 2: Table S1). Patterns resembling specific diseases like tuberculosis, malaria or amoebiasis or processes like DNA replication or mismatch repair are not listed here. Pairwise correlations with MS4A7 (*p* = 3.6x10^−14^), LY96 (*p* = 9.1x10^−19^), MNDA (*p* = 5.6x10^−18^), CSF2RB (*p* = 6.9x10^−12^), and CD68 (*p* = 3,1x10^−6^) also showed correlation with CD73 expression. Moreover, phenotypical markers for myeloid cells (TAM or MDSC) [47] show a significant correlation with both CD39 and CD73 (see Additional file 4: Table S3). Possible explanations for these findings are either expression of ectonucleotidases on myeloid cells in the tumor tissue, or enhanced recruitment of myeloid cells when adenosine is generated.

### Expression of CD39 and CD73 by macrophages from ovarian cancer ascites and in ovarian cancer tissue

**Fig. 2:**
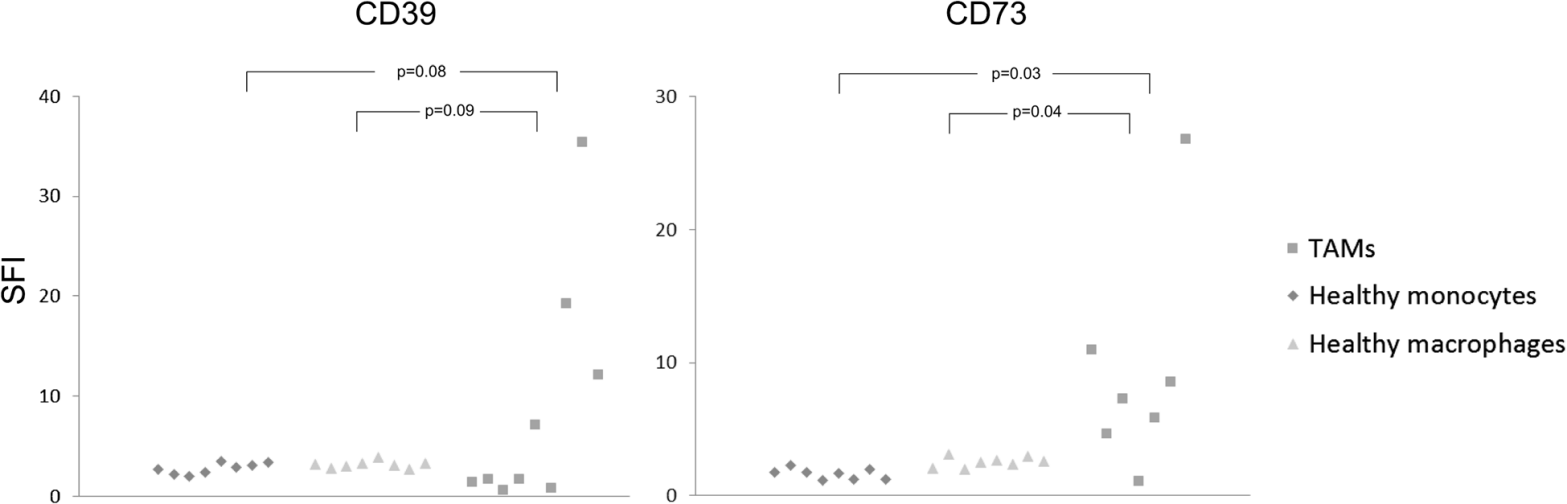
Ectonucleotidases CD39 and CD73 are expressed on CD14^+^ cells from OvCA ascites. Primary CD14^+^ cells (TAM) were isolated from OvCA ascites via magnetic cell sorting (*n* = 9). For control purposes, macrophages and monocytes from healthy donors (*n* = 8) and peritoneal macrophages from patients with benign disease (*n* = 8) were obtained and analyzed. For statistical comparison, unpaired two-sided Student’s *t* test was used

**Fig. 3:**
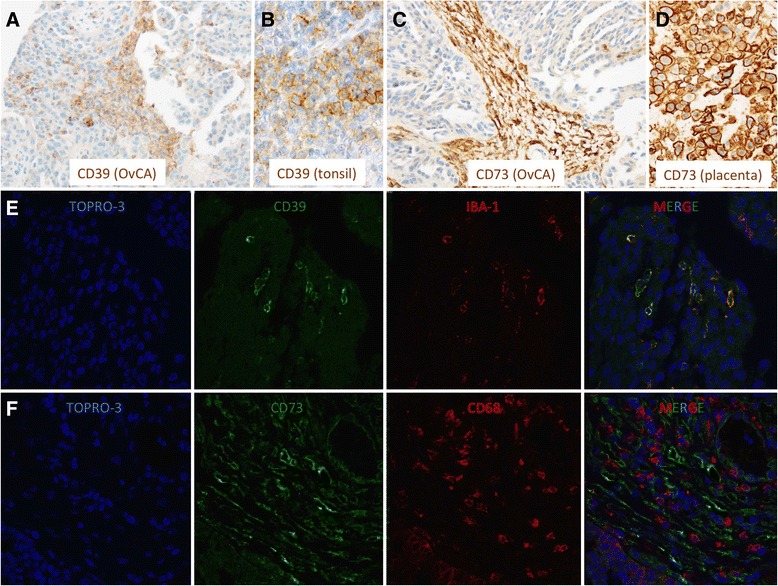
CD39 is expressed on TAM while CD73 is strongly related to tumor stroma in OvCA. Representative immunohistochemical stainings for (**a**-**b**) CD39 and (**c**-**d**) CD73 showing that (**a**) CD39 is heterogeneously expressed on cells within the tumor parenchyma (**b**: tonsil serving as positive control) while (**c**) CD73 expression is largely restricted to the tumor stroma (**d**: placenta serving as positive control). (**e**-**f**) Immunfluorescent double stainings for (**e**) CD39 and IBA-1 as well as (**f**) CD73 and CD68 revealed a considerable co-expression of the macrophages markers with (**e**) CD39 but not with (**f**) CD73

CD14^+^ cells were isolated via magnetic cell separation from fresh ascites samples of OvCA patients. These monocyte-derived cells which are likely to represent ex situ-TAMs were found to express both ectonucleotidases (CD39: 3/3 and CD73: 2/3; Fig. 2). In contrast, macrophages and monocytes from healthy donors as well as peritoneal macrophages from patients with benign disease showed negligible expression of both CD39 and CD73 (Fig. 2). With regard to solid ovarian cancer tissue, expression of CD39 and CD73 in ovarian cancer stroma was already shown in our previous study (Häusler et al., 2011). Aiming at a more precise cellular attribution of ectonucleotidase expression on tumor-associated myeloid cells, 10 OvCA cases, 1 healthy ovary and 1 placenta were investigated. Besides strong endothelial CD39 and CD73 stainings in both normal and neoplastic tissue regions on all OvCA samples, CD39 was clearly detectable on tumor-infiltrating immune cells in 7/10 samples with particularly strong stainings in two cases. Tumor cells, however, were only focally positive for CD39 with strong signals in 1/10 and weak stainings in 9/10 OvCA samples (Fig. 3a; human tonsil serving as positive control as shown in Fig. 3b). In the tumor stroma, CD39 showed a clear signal in 1/10, but no more than weak focal stainings in 9/10 OvCA samples whereas CD73 was prominently expressed in all cases (Fig. 3c; human placenta serving as positive control as shown in Fig. 3d). On tumor cells, CD73 was highly expressed in 1/10 cases, still prominently in 4/10 tissue samples, weak on 3/10 sections and absent in 2/10 cases. Further CD73 expression was detected on tumor-infiltrating immune cells in 6/10 cases. To further determine a potential expression of CD39 and CD73 on myeloid cells, immunofluorescent double staining with myeloid markers were performed revealing a co-localization of CD39 with the macrophage marker IBA-1 (Fig. 3e). CD73, in contrast, was most prominently expressed on fibroblast-like tumor stromal cells without showing a co-localization with cells expression the myeloid marker CD68 (Fig. 3f). Having found that TAMs from ascites express CD39 and CD73 whereas myeloid cells in solid tumor tissue express no CD73 but moderate levels of CD39, ectonucleotidase levels on TAM or MDSC seems to be context-dependent and, likely, inducible. This, however, appears insufficient to explain the strong correlation between CD39, CD73 and myeloid markers found by gene expression analysis. Consequently, we wondered whether adenosine generated via ectonucleotidases on stromal or on tumor cells could attract myeloid cells towards the tumor tissue.

### OvCA cells increase the migration of myeloid precursor cells by CD39- and CD73-dependent generation of adenosine

**Fig. 4:**
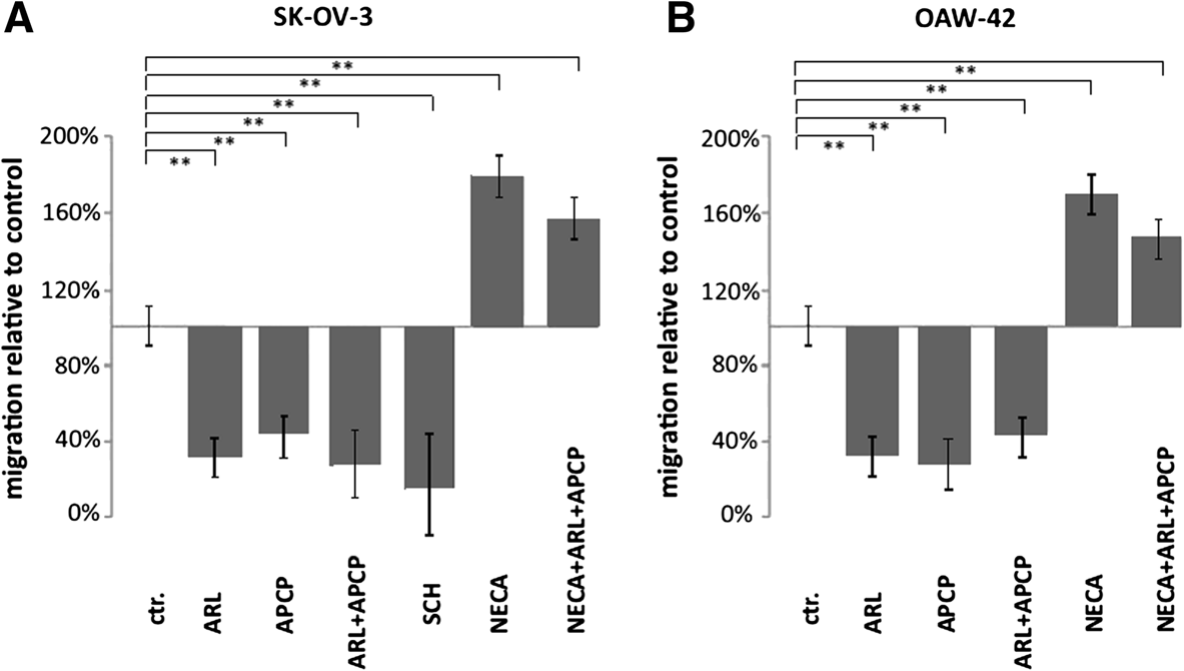
CD39 and CD73 activity on OvCA cells promote monocyte migration in transwell chambers. Primary human monocytes were placed in the upper inserts of transwell plates while SK-OV-3 (**a**) or OAW-42 (**b**) OvCA cells were seeded in the respective bottom compartments. To explore a potential influence of ectonucleotidases, CD39 activity in tumor cells was inhibited by 100 μM ARL67156 whereas CD73 was inhibited with 100 μM α,β-methyleneadenosine-5′-diphosphate (APCP). Equal amounts of solvent (DMSO) were added to the otherwise untreated controls. To exclude unwanted effects of the inhibitors on migrating monocytes, ovarian cancer cells were pre-incubated with the inhibitors for 30 min before being washed with PBS. To assess the effect of adenosine on monocyte migration directly, positive controls with the metabolically stable adenosine receptor agonist adenosine-5′-N-ethylcarboxamide (NECA) (used at 100 nM) were also included, both in the absence and in the presence of APCP and ARL67156 (*n* = 3, * indicates *p* < 0.05, ** denotes *p* < 0.01, as assessed by unpaired Student’s *t*-test). As additional control, A2A receptor signalling was blocked during the assay by SCH58261 (in **a**). Under all conditions, migration of monocytes through the 8 μm wide and 10 μm thick pores was analyzed after 4 h by staining transmigrated cells for CD11c followed by flow cytometric analysis. Tumor cells from the bottom compartment were identified by co-staining for EpCAM expression

To analyze the migration behavior of human blood-borne myeloid cells, CD14^+^ monocytes were isolated from healthy volunteers and placed in the upper inserts of transwell plates. After 4 h of co-incubation with SK-OV-3 or OAW-42 cells in the corresponding bottom compartments, migration of monocytes through the transwell-pores towards the OvCA cells was determined by flow cytometry. Unfortunately, the difficulties in measuring the easily degraded nucleoside adenosine did not allow concomitant determination of adenosine levels during the assay. However, based on our reporter gene assay conditions adenosine levels would typically be in the range from 1.1-1.7 μM for SK-OV-3 and 1.7-4.3 μM for OAW-42 cells. Under these conditions, pre-treatment of the tumor cells with the selective CD39- or CD73-inhibitors ARL67156 or APCP did not affect their viability, but reduced monocyte migration by more than half, as compared to the solvent control. A similar effect was obtained by adding the A2A receptor inhibitor SCH58261 to the monocytes in the upper compartment. Conversely, when the metabolically stable adenosine receptor agonist NECA was applied, monocyte migration was increased by approximately two third (Fig. 4). Importantly, addition of NECA overruled the inhibition of CD39 and CD73 which indicates that the impaired migration was not due to direct effects of the inhibitors on the monocytes but rather to the reduced availability of adenosine (Fig. 4). While no evidence was obtained for enhanced chemokinesis in the presence of NECA, the co-culture setting does not allow to distinguish between direct chemotaxis towards adenosine or a more indirect effect by which adenosine might enhance cell migration towards another tumor-derived chemokine. Still, to screen whether adenosine might induce chemokine or chemokine receptor expression on monocytes, we performed an RT^2^ Profiler PCR array for human chemokines & receptors followed by qRT-PCR for validation of individual candidate molecules (Additional file 1). This, however, showed only CCL28 and CXCL3/GRO-γ to be induced by NECA after 3 h. As CCR10 which is the receptor for CCL28 could not be detected, CCL28 is most unlikely to mediate the observed migratory effect. CXCL3/GRO-γ has, however, been described to affect monocyte differentiation and proliferation without altering their migration behavior [48, 49]. Thus, while an indirect or secondary effect on migration cannot be excluded, there is currently no evidence for this. Moreover, the experimental data show that irrespective of the underlying mechanism adenosine can attract monocytes towards tumor cells.

### M2-macrophages polarized by coculture with OvCA cells upregulate CD39 and CD73 to levels also observed in CD14^+^ cells from ovarian cancer ascites

**Fig. 5:**
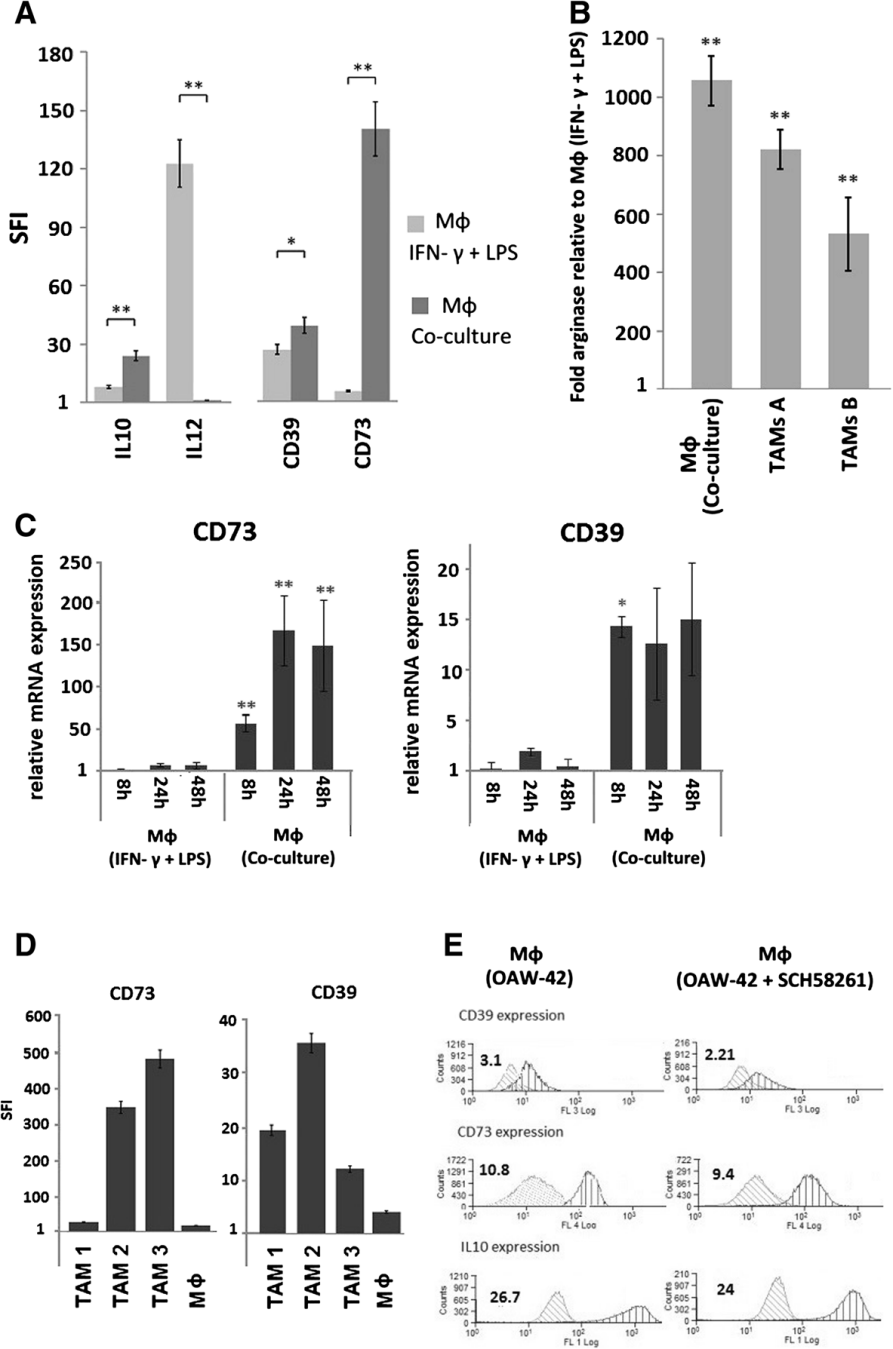
In vitro induced TAM-like macrophages show M2-polarization, arginase activity and ectonucleotidase expression. Peripheral blood monocytes were obtained from healthy volunteers by gradient centrifugation and subsequent adherence enrichment. Monocytes were then matured in Lumox dishes for 9 days to obtain mature macrophages (MФ). Macrophage polarization was achieved either with IFN-γ [1 μg/ml] and LPS [10 μg/ml] (MФ (IFN-γ + LPS)) or by coincubation with OAW-42 OvCA cells (MФ (Co-culture)). **a** The obtained MФ (IFN-γ + LPS) (light grey) and MФ (Co-culture) (dark grey) were analyzed by FACS for intracellular IL-10 and IL-12 levels. To obtain specific fluorescence intensity (SFI) values, the median fluorescence values obtained with fluorochrome-conjugated specific antibodies were divided by the median fluorescence values obtained with identically labeled irrelevant isotype-matched control antibodies (*n* = 3, * for *p* < 0.05, ** for *p* < 0.01). **b** CD14^+^ cells (TAM) were isolated from malignant tumor tissue. In addition, mature macrophages from a healthy donor were polarized as in **a**. To determine arginase activity by MФ (IFN-γ + LPS) and MФ (Co-culture) as well as by TAM, arginine conversion was assessed by measuring the resulting urea in a colorimetric assay. Significance levels were determined by two-sided, unpaired Student’s *t*-test. *p*-values < 0.01 were considered as highly significant (**). **c** Macrophages were prepared and polarized as in **a**. 8 h, 24 h and 48 h after the polarizing conditions had been applied, RNA was isolated, reverse-transcribed to cDNA and analyzed for CD39 and CD73 transcript levels using SybrGreen-based RT-PCR. 18S rRNA content was determined for normalization and relative CD39 and CD73 mRNA levels were calculated by the ΔΔC_t_ method using non-polarized macrophages as reference. **d** Using MФ (IFN-γ + LPS) and MФ (Co-culture) as in **c**, CD39 and CD73 surface expression was assessed by flow cytometry. **e** Macrophages were polarized with OAW-42 cells for 24 h in the presence or absence of the A2A adenosine receptor inhibitor SCH58261 before IL-10, CD39 and CD73 expression were analysed by flow cytometry. Comparable data for primary TAMs isolated from OvCA ascites are shown in Fig. 2

Immunosuppressive myeloid cells with a phenotype resembling tumor-associated macrophages (TAMs) can be induced by co-incubation of monocytes with OvCA cells [43]. Having found that OvCA cell-derived adenosine attracts monocytes towards the tumor cells, we wanted to confirm that these monocytes polarize to an M2-like phenotype which is characteristic for immunosuppressive TAM. Therefore, mature macrophages from healthy donors were co-incubated with OAW-42 cells in a transwell setting where macrophage migration to the lower chamber was precluded by the narrow pore-diameter of the transwell membrane. After 48h of co-culture, macrophages were analysed by intracellular flow cytometry for expression of IL-10 and IL-12. As opposed to M1-polarized macrophages that had been generated from monocytes in the presence of IFN-γ and LPS, co-cultured macrophages displayed high levels IL-10 and low levels of IL-12 confirming their M2-polarization (Fig. 5a).

High arginase activity is also related to a macrophage phenotype promoting tumor growth [50]. Consequently, co-cultured macrophages were further characterized using a colorimetric assay for arginase activity. This confirmed a higher enzymatic activity in the M2 polarized macrophages as well as in TAMs isolated from fresh ascites of OvCA patients (Fig. 5b).

Similar to recent reports on murine TAMs [39–41], human macrophages which had been co-cultured in vitro with ovarian cancer cells (OAW-42) also showed high expression of CD39 and CD73, both on mRNA (Fig. 5c) and on protein level (Fig. 5d). Acquisition of this phenotype was found to be independent of signaling via the A2A adenosine receptor since induction of CD39, CD73 and IL-10 also occurred when SCH58261 was present in the co-culture (Fig. 5e). In M1 macrophages, however, CD39 and CD73 surface levels were much lower (compare Fig. 5a). As this resembled the in vivo situation observed on TAMs from OvCA ascites shown in Fig. 2, we decided to further explore the functional consequences of ectonucleotidase expression on TAM-like M2 macrophages (“in vitro TAMs”).

### Increased CD39 and CD73 expression on in vitro generated TAM-like M2 macrophages translates into higher levels of biologically active adenosine

**Fig. 6:**
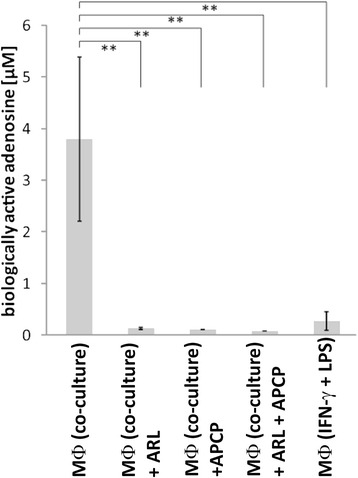
Macrophages from in vitro co-culture with OvCA cells generate adenosine via CD39 and CD73. Human macrophages were generated and polarized in transwell coculture with OAW-42 OvCA cells. For control purposes, M1 macrophages were induced with LPS (10 μg/ml) and IFN-γ (1 μg/ml). After coculture with ADORA2A-overexpressing, RIP1-CRE transfected sensor cells, a luciferase-based reporter assay was performed to determine production of biologically active adenosine [45]. To confirm that adenosine generation depends on ectonucleotidase activity, CD39 was blocked with ARL67156 (MФ (Co-culture) + ARL) during coculture. Likewise, CD73 was inhibited by APCP (MФ (Co-culture) + APCP) and to inactivate both ectonucleotidases, ARL67156 and APCP were also combined (MФ (Co-culture) + ARL + APCP). To the control groups (MФ (Co-culture) and MФ (IFN-γ + LPS)), only the solvent DMSO was added (*n* = 3). Significance levels were determined by Student’s *t*-test. *p*-values < 0.01 were considered as highly significant (**)

To investigate whether the high ectonucleotidase expression on M2 macrophages results in increased levels of biologically active adenosine, a luciferase-based adenosine reporter assay was performed as previously described [45]. In line with the observed expression of adenosine-generating enzymes, pro-inflammatory M1-polarized macrophages produced only low amounts of adenosine (0.27 μM ± 0.18 μM) whereas the TAM-like or M2-polarized macrophages generated significantly higher levels of the immunosuppressive nucleoside (on average 3.8 μM, range: 1.6-5.4 μM) (Fig. 3). In the presence of specific inhibitors for CD39 (ARL 67156) and CD73 (APCP), adenosine production was almost abrogated (Fig. 6).

### In vitro polarized M2 macrophages suppress CD4^+^ T cell proliferation via adenosine production

**Fig. 7:**
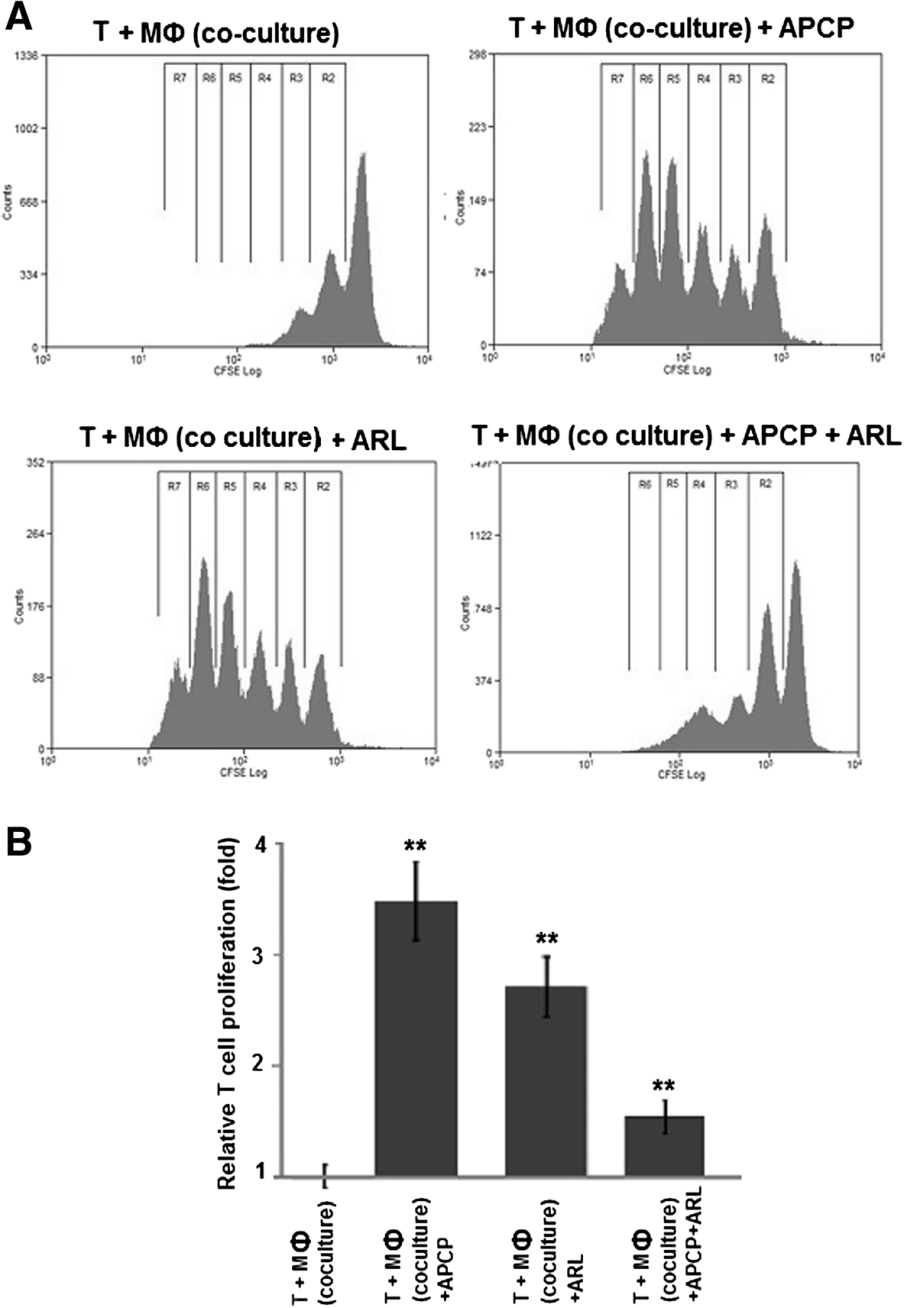
“TAM-like” macrophages suppress CD4^+^ T cell proliferation via a CD39 and CD73-dependent mechanism. Polarized macrophages were generated in co-culture with OAW-42 OvCA cells whereas M1 macrophages were induced by treatment with LPS and IFN-γ. CD4^+^ T cells were isolated from healthy volunteers and stained with CFSE before activation with plate-bound anti-CD3 and soluble anti-CD28 antibodies. Activated CFSE^+^ CD4^+^ T cells were then co-incubated with M2 macrophages in the absence (T + MФ (Co-culture)) or presence of CD39 or CD73-inhibitors ARL67156 (T + MФ (Co-culture) + ARL) or, respectively, APCP (T + MФ (Co-culture) + APCP), or a combination of both (T + MФ (Co-culture) + ARL + APCP). T cell proliferation was determined by flow cytometry via CFSE dilution, as described previously [7] (*n* = 3, ** indicates *p* < 0.01 as assessed by unpaired, two-sided Student’s *t*-test). While a representative histogram is shown in **a**, mean values from three independent experiments are depicted in (**b**)

To test the functional relevance of increased adenosine generation by M2 macrophages, their effect on CD4^+^ T cell proliferation was investigated in coculture experiments with CD4^+^ T cells which had been stimulated with agonistic antibodies to CD3 and CD28. In this setting, TAM-like M2 macrophages decreased CD4^+^ T cell proliferation by >50 % (52 % on average, range: 47–56 %) when compared to M1 macrophages (a representative example is shown in Fig. 7a, pooled data from 3 independent experiments are displayed in Fig. 7b). To specifically block ectonucleotidase activity on M2 macrophages, ARL67156, APCP, or both were added. Compared to solvent controls, CD4^+^ T cell proliferation was increased about 3.5-fold [range: 3.1 - 3.8-fold] by blockade of CD39 with ARL67156. Inhibition of CD73 by APCP enhanced T cell proliferation by a factor of 2.7 [range: 2.4 – 3.0] (Fig. 7). Considering that both APCP and ARL67156 already abrogated adenosine production when used on their own (compare Fig. 6), no further improvement could be expected by their combination. Instead, a somewhat diminished effect was observed which may be due to slight toxic effects caused by the simultaneous use of both inhibitors (Fig. 7).

## Discussion

In hypoxic tissues or after chemotherapeutic treatments, dying tumor cells are abundant. ATP released by these cells [7, 51–53] constitutes an immunological danger signal which can activate dendritic cells [35]. ATP can, however, be rapidly converted to adenosine which is known to suppress CD4^+^ T, CD8^+^ T and NK cells [7, 14, 54]. In fact, we and others have already shown that ovarian cancer cells and tissues can express high levels of the ectonucleotidases CD39 and CD73 which enzymatically convert free extracellular ATP to adenosine. CD39 (which catalyzes the degradation of pro-inflammatory ATP to AMP) even showed a highly tumor-specific expression pattern in the female reproductive tract. CD73 (which generates adenosine from AMP) was likewise significantly overexpressed in tumor tissues [7]. Moreover, strategies directed at the inhibition of CD73 (and thus at the prevention of adenosine generation), have shown impressive results in animal models of breast and ovarian cancer [8, 10, 55]. Nevertheless, both positive and negative correlations between CD39 and CD73 expression and outcome have been described [56–59]. The desirable analysis whether local adenosine levels affect prognosis is unfortunately precluded by the short half-life of adenosine. Gene expression analysis, however, suggests that combined overexpression of CD39 and CD73 is associated with a trend towards poor survival (Fig. 1, *p* = 0.062, *n* = 347 patients).

A clear correlation has, in contrast, been found for infiltration of ovarian tumors with myeloid cells and rapid tumor progression, metastasis and poor prognosis [24–26]. As human studies are confined to readily available biological materials, we used monocytes as easily available human myeloid cells and co-cultured them with ovarian cancer cells, thereby mimicking the maturation and M2 differentiation of myeloid cells in the tumor microenvironment [43]. CD14^+^ cells derived from ascites (Fig. 2) were used ex vivo to corroborate the in vitro findings. Based on these cellular model systems, we could show that OvCA-derived adenosine exerts direct or indirect chemotactic effects on human monocytes (Fig. 4) and is thus likely to attract myeloid precursor cells towards the tumor tissue. Moreover, in a microenvironment that is shaped by OvCA cells, human monocytes differentiate into M2-polarized macrophages or TAM (Fig. 5) which express IL-10 rather than IL-12 and show arginase activity. While signalling via the A2A adenosine receptor is not required for M2 polarization, M2 macrophages express significant levels of the adenosine-generating ectonucleotidases CD39 and CD73, both on mRNA and protein level (Fig. 6). This is in line with recent reports on CD73 expression by murine MDSC which were also shown to suppress immune responses in an adenosine-dependent way [19, 39, 40]. Moreover, CD14^+^ cells isolated from OvCA ascites also showed much higher levels of CD39 and, in particular, CD73 than macrophages from non-malignant control ascites (Fig. 2). Interestingly, similar findings were also reported in a most recent publication describing an IL-27 dependent induction of CD39 on macrophages in ovarian cancer patients [60]. Using a reporter gene assay developed in our laboratory [45], we could then confirm that the amount of biologically active adenosine generated by M2 macrophages was similar to the levels produced by OvCA cells [7], i.e., in the low micromolar range (Fig. 4) and thus 30-60-fold higher than the already immunosuppressive levels from T_reg_ [45]. Consequently, co-culture experiments also confirmed that M2 macrophages exert strong anti-proliferative effects on activated CD4^+^ T cells. Importantly, T cell proliferation was largely restored when CD39 or CD73 were blocked by ARL67156 or APCP, respectively, thereby confirming the pivotal role of adenosine in the observed T cell inhibition (Fig. 7).

While these data show that ectonucleotidase expression by myeloid cells may be functionally relevant, an attribution to either MDSC or TAM was not attempted in our study. As monocytic MDSC which are predominant in tumor tissues [28, 29] tend to rapidly differentiate into TAM [61], discrimination between these cell types is difficult and gradual. Polymorphonuclear MDSC, in contrast, do not develop into TAM and should thus be seen as different cell population [62]. Using common myeloid markers, immunohistochemical analysis of solid ovarian tumor tissue failed to show a co-localization of CD73 with CD68 (Fig. 3f). CD39, in contrast, could be co-stained with the macrophage marker IBA-1 (Fig. 3e). While the limited sensitivity of immunohistochemical analysis may also contribute to the apparent lack of CD73 on TAM or MDSC in solid ovarian cancer tissue, expression of ectonucleotidases on myeloid cells may also be context-dependent and could require e.g., inflammatory stimuli as found in ascites or under in vitro conditions. Still, both CD39 and CD73 were detected on both tumor and stromal cells in ovarian cancer tissue and prominent expression was found on endothelial cells from the tumor vasculature. Infiltrating immune cells may thus be already exposed to adenosine during transmigration into the tissue. Further, recruitment of macrophage precursor cells via adenosine-dependent chemotaxis may be particularly efficient when adenosine is generated at the tumor-vessel interface. The partial co-expression between CD39 and IBA-1^+^ cells is certainly insufficient to fully account for the strong correlation between macrophage-specific genes and ectonucleotidase expression (Additional file 2: Table S1 and Additional file 3: Table S2) or for the highly significant association between CD39, CD73 and the KEGG data set for antigen processing and presentation (Additional file 4: Table S3). An additional adenosine-dependent recruitment of myeloid cells to the tumor microenvironment may therefore also have contributed to the strong correlations found by unbiased transcriptome analyses from 285 ovarian cancer tissues.

As this study aimed at establishing a link between adenosine and myeloid cells in the tumor microenvironment, the mechanism responsible for the adenosine-dependent recruitment of monocytes towards tumor cells was not investigated in detail. In the literature, diverse effects of adenosine on cell migration have already been reported: While ATP enhances the non-directional motility of monocytes [63], degradation of ATP to adenosine by CD39 was found to promote the chemotactic migration of myeloid cells in a modified Boyden chamber assay. Consequently, chemotaxis of CD39-deficient monocytes/macrophages was impaired [64]. As this effect could also be rescued by addition of NECA, it is fully consistent with our observations made with ARL67156 and NECA. Similar findings on adenosine and cell migration were reported for neutrophils and initially attributed to the involvement of the A2 adenosine receptor [65]. Later, an addtitional and possibly stronger contribution from the A1 adenosine receptor was described [66]. In a more complex system involving a pericellular matrix and smooth muscle cells, adenosine also promoted the migration of monocytes/macrophages and their trapping in the interstitial matrix. This, however, was ascribed to an indirect effect caused by hyaluronic acid synthase being induced via the A2B, A2A and A3 adenosine receptors, with different kinetics for each receptor [67]. Without agreeing on a single mode-of-action, various reports have thus already described direct and indirect pro-migratory effects of adenosine on monocyte. Consistent with its overall anti-inflammatory effect, adenosine may, in contrast, also reduce the migration of pro-inflammatory cell types [68]. This may also apply to activated monocytes and microglia which were reported to migrate less in response to activation of the A3 adenosine receptor [69]. Adenosine was further found to slow the migration of dendritic cells [70] and to inhibit transendothelial migration of conventional T cells via an indirect monocyte-dependent mechanism [71]. While these divergent effects may be due to cell type and activation state-dependent expression patterns of adenosine receptors A1, A2A, A2B and A3, the analysis of receptor distribution is hampered by the lack of suitable antibodies against the individual receptor subtypes. Still, it appears clear that adenosine can affect cell migration via direct and indirect mechanisms involving different adenosine receptors depending on cell type, assay and kinetics.

## Conclusion

Our findings that human monocytes are attracted by adenosine while M2 macrophages can contribute to local immunosuppression via adenosine generation suggest a self-amplifying mechanism: Similar to what has already been described in mice [40] and what needs to be further explored in vivo, tumor-derived adenosine might promote recruitment of further adenosine-generating cells towards the microenvironment in human cancer patients. Infiltration with MDSC or TAM would then be both a consequence and a further source of tumor-derived adenosine. This might further explain the very potent effects observed with anti-CD73 and anti-ADORA2A directed strategies in animal tumor models [8, 10, 55] – and it could show a new strategy to interfere with MDSC, TAM and their tumor-promoting role in ovarian cancer. Importantly, the substances we used to specifically block CD39 and CD73 activity are already being tested in vivo for other diseases: APCP was found to be highly beneficial in a mouse melanoma model [72] whereas the CD39-inhibitor ARL67156 has shown promise for the treatment of hepatic insulin resistance [73]. Thus, such inhibitors might also become available for the clinical treatment of malignant diseases. Based on previous findings and on the data outlined in this manuscript, there certainly is a strong rationale for a potential use of ectonucleotidase inhibitors in the immunotherapeutic treatment of ovarian cancer.

## Abbreviations

(R-)PE, R-phycoerithrin; ADORA2A, adenosine receptor 2A; APCP, α,β-methyleneadenosine-5′-diphosphate; ARL67156, 6-*N*,*N*-Diethyl-D-β,γ-dibromomethyleneATP trisodium salt; CCL, CC-chemokine ligand; CFSE, carboxyfluorescein diacetate succinimidyl ester; CI, confidence interval; CRE, cAMP responsive element; CXCL, CXC-chemokine ligand; DAB, diaminobenzidine; DMSO, dimethyl sulfoxide; ENTPD1, ectonucleoside triphosphate diphosphohydrolase 1; FITC, Fluorescein isothiocyanate; GRO-γ, growth regulated oncogene gamma; hrp, horseradish peroxidase; IBA1, ionized calcium-binding adapter molecule 1 (also known as Allograft inflammatory factor 1 (AIF-1)); IDO, indoleamine-2,3-dioxygenase; KEGG, Kyoto encyclopedia of genes and genomes; LPS, lipopolysaccharide; Luc, firefly (*photinus pyralis*) luciferase; MDSC, myeloid-derived suppressor cells; NECA, adenosine-5′-N-ethylcarboxamide; NK, natural killer (cells); OvCA, ovarian cancer; PBMC, peripheral blood mononuclear cells; RIP1, rat insulin 1 gene promoter; RL, *renilla reniformis* luciferase; ROS, reactive oxygen species; SCH58261, 5-Amino-7-(2-phenylethyl)-2-(2-furyl)-pyrazolo(4,3-e)-1,2,4-triazolo(1,5-c)pyrimidine; TAM, tumor associated macrophages; T_reg_, regulatory T cells

## Additional files

Additional file 1: RT² Profiler PCR array for human chemokines & receptors. (DOCX 184 kb) Additional file 2: Table S1. KEGG pathway analysis using the R2 pathway finder. List of pathways significantly correlated with the expression of CD39 or CD73 and their respective p values. (DOCX 15 kb) Additional file 3: Table S2. Analysis of the antigen processing and presentation pathway and gene-wise correlation with the expression of CD39 (ENTPD1) and CD73 (NT5E). Out of 57 genes linked to this pathway 42 genes show correlation with CD39 and 41 genes correlate with CD73. (DOCX 15 kb) Additional file 4: Table S3. Gene expression correlation analysis. Correlation between CD39 (ENTPD-1) or CD73 (NT5E) and phenotypic markers on human myeloid cells. Gene expression data from 285 ovarian cancer tissues from the AOCS (Australian Ovarian Cancer Study) were screened for genes correlating with the presence of CD39 (ENTPD-1) or CD73 (NT5E). Pairwise correlation analyses indicated positive correlations between almost all phenotypical markers described for MDSC (Talmadge and Gabrilovich, 2013) and the ectoenzymes CD73 and CD39. (DOCX 14 kb)
