# Supplementary material for: Adenosine-generating ovarian cancer cells attract myeloid cells which differentiate into adenosine-generating tumor associated macrophages – a self-amplifying, CD39- and CD73-dependent mechanism for tumor immune escape
Source: J Immunother Cancer. 2016 Aug 16;4:49. doi: 10.1186/s40425-016-0154-9 (PMC4986205; doi:10.1186/s40425-016-0154-9)
Supplement: Additional file 1: — RT² Profiler PCR array for human chemokines & receptors. (DOCX 184 kb) [file 40425_2016_154_MOESM1_ESM.docx]

**Supplemental Experiment: RT² Profiler PCR array for human chemokines & receptors**

To investigate if NECA has an effect on the expression of chemokines or chemokine receptors in monocytes, mRNA expression on CD14^+^ bead-purified monocytes was assessed 3h after NECA treatment in comparison to an untreated control. For each sample, 280ng total RNA were used for cDNA synthesis (RT² First Strand Kit, Qiagen, Hilden, Germany). The RT² Profiler PCR array for human chemokines & receptors and the RT² SYBR Green ROX qPCR mastermix (both from Qiagen) were used according to the manufacturer’s instructions. Assays were run on a StepOnePlus RealTime-PCR cycler (thermofisher, Darmstadt, Germany). For data analysis, the same threshold was applied for each plate and data were analyzed in the Qiagen Analysis Webportal.


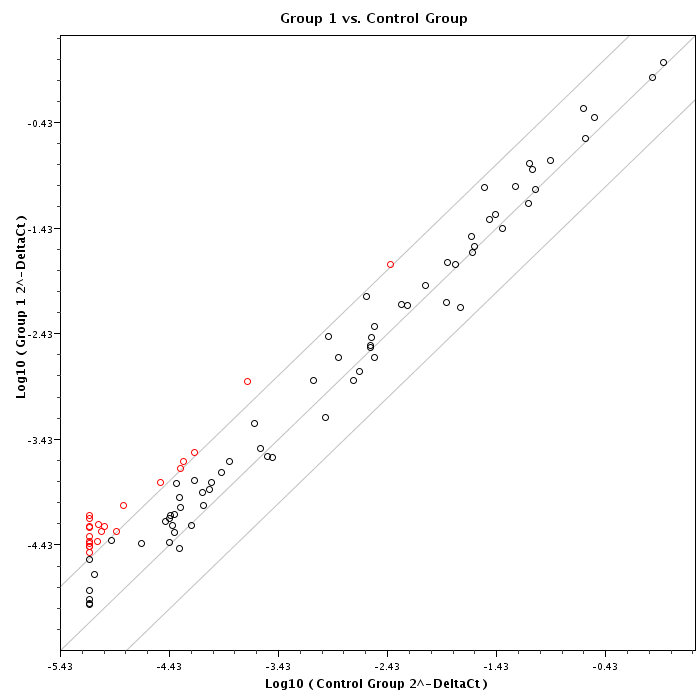

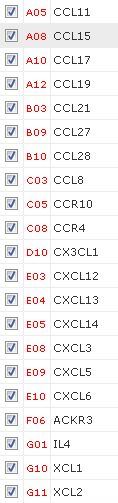

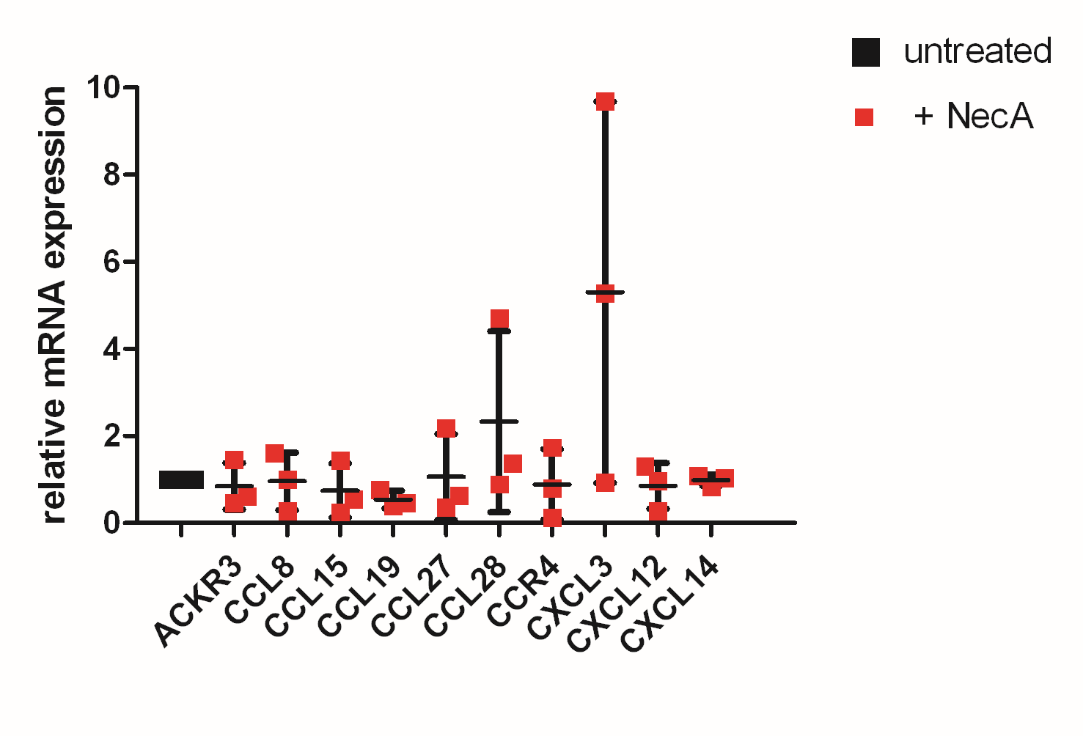


**Figure S1: Treatment of monocytes with NECA induces changes in chemokine and chemokine receptor mRNA expression.** **Upper panel.** Primary human monocytes were isolated from peripheral blood and treated for 3 h with 100ng NECA (group 1, y-axis) or with the equivalent volume of DMSO (control group, x-axis). RNA expression was analyzed using the RT² Profiler PCR Array. **Lower panel.** Primary human monocytes were isolated from peripheral blood of three donors and treated with 100ng NECA (red squares) or the equivalent volume of DMSO (black squares) for 3h. RNA expression was analyzed via ReatTime-PCR.

When monocytes were treated with NECA the screening assay suggested that three receptors (ACKR3, CCR4 and CCR10) and 18 ligands (CCL8, CCL11, CCL15, CCL17, CCL19, CCL21, CCL27, CCL28, CX3CL1, CXCL3, CXCL5, CXCL6, CXCL12, CXCL13, CXCL14, IL-4, XCL1, XCL2) were upregulated in comparison to the untreated control (Figure R1). For eight of these ligands, however, no effect on monocytes had been reported (CCL11, CCL17, CX3CL1, CXCL5, CXCL6, CXCL13, XCL1, XCL2). These hits were thus omitted. Likewise, IL-4 was not further investigated since it does not affect monocyte migration. To validate the remaining candidates, the experiment was repeated with three donors and analyzed by qRT-PCR. Primer sequences are provided below.

With regard to chemotactic receptors, we did not observe an effect on ACKR3 and CCR4 mRNA expression when monocytes were treated with NECA (Figure R2). CCR10 mRNA expression was detected in freshly isolated peripheral blood mononuclear cells, but not in monocytes. Functionally, CCR10 is unlikely to affect monocyte function or migration.

While no changes in mRNA expression were observed for CCR3, CCL8, CCL15, CCL19, CCL27, CXCL12 and CXCL14, CCL21 was not detected at all. CCL28, in contrast, was found to be induced by adenosine. Its receptor CCR10 was, however, not detected on monocytes. Moreover, we are not aware of any reports linking CCL28 and CCR10 to monocyte migration.

The chemokine most strongly upregulated was CXCL3, also called growth regulated oncogene gamma (GRO-γ). CXCL3 has been described to affect monocyte differentiation and proliferation without altering their migration behavior [[1](#_ENREF_1), [2](#_ENREF_2)]. Thus, we cannot fully exclude indirect effects. The chemokines induced by the adenosine analogue NECA, i.e. CCL28 and CXCL3, are unlikely to be responsible for the observed pro-migratory effects.

1. Chen HW, Chen HY, Wang LT, Wang FH, Fang LW, Lai HY, Chen HH, Lu J, Hung MS, Cheng Y, et al: **Mesenchymal stem cells tune the development of monocyte-derived dendritic cells toward a myeloid-derived suppressive phenotype through growth-regulated oncogene chemokines.** *J Immunol* 2013, **190:**5065-5077.

2. Smith DF, Galkina E, Ley K, Huo Y: **GRO family chemokines are specialized for monocyte arrest from flow.** *Am J Physiol Heart Circ Physiol* 2005, **289:**H1976-1984.

Primer Sequences:

ACKR3 frw: 5´- CTGCGTCCAACAATGAGACCTA-3´ (777-798),

ACKR3 rev: 5´- CCGATCAGCCACTCCTTGAT-3´ (846-827);

CCL8 frw: 5´- CCCTTGCCCTCCAAGATGA -3´ (441-459),

CCL8 rev: 5´- CTGAAAGTGGCTGCCATGAG -3´ (508-489);

CCL15 frw: 5´- TGCTTGTTGCTGTCCTTGGA-3´ (581-600),

CCL15 rev: 5´- GGAAGCTTTGACATCATTAACTCTGT-3´ (656-631);

CCL19 frw: 5´- CCTCAGCCTGCTGGTTCTCT-3´ (156-175),

CCL19 rev: 5´- CAGCAGTCTTCAGCATCATTGG-3´ (227-206);

CCL21 frw: 5´- CCAAGCTTAGGCTGCTCCAT-3´ (258-277),
CCL21 rev: 5´- TGCACATAGCTCTGCCTGAGA-3´ (329-309);

CCL27 frw: 5´- AGCCTCCTGCTGCTGTCATT-3´ (84-103),

CCL27 rev: 5´- TGCTGGGTGGCAGTAGGAA-3´ (150-132);

CCL28 frw: 5´- CGTCCACGGCCTTAAAGAAG-3´ (199-218),

CCL28 rev: 5´- TGTGAAACCTCCGTGCAACA-3´ (273-254);

CCR4 frw: 5´- CAATACTGTGGGCTCCTCCAA-3´ (1156-1176),

CCR4 rev: 5´- TCCATGGTGGACTGCGTGTA-3´ (1226-1207);

CCR10 frw: 5´- GGCCACAGAGCAGGTTTCC-3´ (16-34),

CCR10 rev: 5´- CATCGGCCTTGTAGCAAAGC-3´ (110-91);

CXCL3 frw: 5´- AAGAAGCTTATCAGCGTATCAT -3´ ,

CXCL3 rev: 5´- AATAAGTAGAACCCTCGTAAGAAA -3´ ;

CXCL12 frw: 5´- CCGTCAGCCTGAGCTACAGAT-3´ (117-137),

CXCL12 rev: 5´- GACGTTGGCTCTGGCAACA-3´ (181-163);

CXCL14 frw: 5´- GACGTGAAGAAGCTGGAAATGA-3´ (613-634),

CXCL14 rev: 5´- CGCTCTTGGTGGTGATGATAAC-3´ (688-667);
