# Supplementary material for: Adenosine-generating ovarian cancer cells attract myeloid cells which differentiate into adenosine-generating tumor associated macrophages – a self-amplifying, CD39- and CD73-dependent mechanism for tumor immune escape
Source: J Immunother Cancer. 2016 Aug 16;4:49. doi: 10.1186/s40425-016-0154-9 (PMC4986205; doi:10.1186/s40425-016-0154-9)
Supplement: Additional file 2: Table S1. — KEGG pathway analysis using the R2 pathway finder. List of pathways significantly correlated with the expression of CD39 or CD73 and their respective p values. (DOCX 15 kb) [file 40425_2016_154_MOESM2_ESM.docx]

## Supplemental Table 1

| **CD39 ( ENTPD1)** | | **CD73 (NT5E)** | |
| --- | --- | --- | --- |
| **Pathway** | **p** | **Pathway** | **p** |
| Osteoclast differentiation | **6.1x10^-09^** | Focal adhesion | **1.2x10^-05^** |
| Ribosome | **7.9x10^-08^** | Osteoclast differentiation | **1.7x10^-05^** |
| Phagosome | **9.7x10^-07^** | Phagosome | **2.8x10^-05^** |
| **Antigen processing and presentation** | **1.5x10^-05^** | Cytokine-cytokine receptor interaction | **4.3x10^-05^** |
| Cell adhesion molecules | **4.4x10^-05^** | Cell cycle | **1.1x10^-04^** |
| Intestinal immune network for IgA production | **5.4x10^-05^** | Proteoglycans in cancer | **1.1x10^-04^** |
| Lysosome | **6.1x10^-05^** | Natural killer cell mediated cytotoxicity | **1.7x10^-04^** |
| TNF signaling pathway | **1.9x10^-04^** | Pyrimidine metabolism | **3.2x10^-04^** |
| NF-κB signaling pathway | **2.0x10^-04^** | DNA replication | **3.9x10^-04^** |
| NOD-like receptor signaling pathway | **3.7x10^-04^** | ECM-receptor interaction | **4.7x10^-04^** |
| Primary immunodeficiency | **9.0x10^-04^** | TNF-signaling pathway | **4.9x10^-04^** |
|  |  | Chemokine signaling pathway | **8.6x10^-04^** |
| ECM-receptor interaction | **1.6x10^-03^** | Base excision repair | **9.6x10^-04^** |
| Natural killer cell mediated cytotoxicity | **4.4x10^-03^** | **Antigen processing and presentation** | **1.6x10^-03^** |
| Toll-like receptor signaling pathway | **4.9x10-^03^** | Leukocyte transendothelial migration | **1.9x10^-03^** |
| TGF-beta signaling pathway | **7.5x10^-03^** | Hematopoietic cell lineage | **2.0x10^-03^** |
| Cytokine-cytokine receptor interaction | **7.9x10^-03^** | Regulation of actin cytoskeleton | **3.3x10^-03^** |
| Leukocyte transendothelial migration | **8.3x10^-03^** | Prostate cancer | **3.5x10^-03^** |
|  |  | Pathways in cancer | **3.8x10^-03^** |
|  |  | T cell receptor signaling pathway | **5.6x10^-03^** |
|  |  | B cell receptor signaling pathway | **7.8x10^-03^** |
|  |  | PI3K-Akt signaling pathway | **8.3x10^-03^** |
|  |  | MicroRNAs in cancer | **8.7x10^-03^** |

**Supplemental Table 1:** **KEGG pathway analysis using the R2 pathway finder**. List of pathways significantly correlated with the expression of CD39 or CD73 and their respective p values.
