# Supplementary material for: Adenosine-generating ovarian cancer cells attract myeloid cells which differentiate into adenosine-generating tumor associated macrophages – a self-amplifying, CD39- and CD73-dependent mechanism for tumor immune escape
Source: J Immunother Cancer. 2016 Aug 16;4:49. doi: 10.1186/s40425-016-0154-9 (PMC4986205; doi:10.1186/s40425-016-0154-9)
Supplement: Additional file 3: Table S2. — Analysis of the antigen processing and presentation pathway and gene-wise correlation with the expression of CD39 (ENTPD1) and CD73 (NT5E). Out of 57 genes linked to this pathway 42 genes show correlation with CD39 and 41 genes correlate with CD73. (DOCX 15 kb) [file 40425_2016_154_MOESM3_ESM.docx]

|  | **CD39 (ENTPD-1)** | | **CD73 (NT5E)** | |
| --- | --- | --- | --- | --- |
| **HUGO** | **R** | **p** | **R** | **p** |
| B2M | 0.363 | 2.7x10^-10^ | 0.286 | 9.1x10^-07^ |
| CD4 | 0.538 | 9.4x10^-23^ | 0.299 | 2.7x10^-07^ |
| CD74 | 0.435 | 1.3x10^-14^ | 0.415 | 2.5x10^-13^ |
| CD8A | 0.495 | 4.9x10^-19^ | 0.275 | 2.5x10^-06^ |
| CD8B | 0.365 | 2.0x10^-10^ | 0.138 | 0.02 |
| CIITA | 0.282 | 1.3x10^-06^ | 0.259 | 9.6x10^-06^ |
| CTSB | 0.456 | 5.0x10^-16^ | 0.405 | 1.2x10^-12^ |
| CTSS | 0.564 | 2.7x10^-25^ | 0.343 | 2.7x10^-09^ |
| HLA-A | 0.399 | 2.7x10^-12^ | 0.266 | 5.1x10^-06^ |
| HLA-B | 0.403 | 1.4x10^-12^ | 0.329 | 1.3x10^-08^ |
| HLA-C | 0.393 | 6.1x10^-12^ | 0.280 | 1.6x10^-06^ |
| HLA-DMA | 0.437 | 1.0x10^-14^ | 0.360 | 3.7x10^-10^ |
| HLA-DMB | 0.475 | 1.8x10^-17^ | 0.205 | 4.9x10^-04^ |
| HLA-DOA | 0.361 | 3.5x10^-10^ | 0.343 | 2.7x10^-09^ |
| HLA-DOB | 0.315 | 5.3x10^-08^ | 0.123 | 0.04 |
| HLA-DPA1 | 0.457 | 4.4x10^-16^ | 0.446 | 2.5x10^-15^ |
| HLA-DPB1 | 0.509 | 3.3x10^-20^ | 0.399 | 2.6x10^-12^ |
| HLA-DQA1 | 0.182 | 2.1x10^-03^ | 0.048 | 0.42 |
| HLA-DQB1 | 0.475 | 1.9x10^-17^ | 0.421 | 1.1x10^-13^ |
| HLA-DRA | 0.485 | 3.1x10^-18^ | 0.402 | 1.8x10^-12^ |
| HLA-DRB1 | 0.452 | 9.3x10^-16^ | 0.437 | 1.0x10^-14^ |
| HLA-DRB4 | 0.182 | 2.0x10^-03^ | 0.228 | 1.1x10^-04^ |
| HLA-E | 0.455 | 5.3x10^-16^ | 0.311 | 8.2x10^-08^ |
| HLA-F | 0.453 | 7.9x10^-16^ | 0.260 | 8.7x10^-06^ |
| HLA-G | 0.413 | 3.4x10^-13^ | 0.243 | 3.4x10^-05^ |
| HSP90AA1 | -0.122 | 0.04 | 0.113 | 0.08 |
| HSP90AB1 | -0.219 | 2.0x10^-04^ | -0.167 | 4.7x10^-03^ |
| HSPA5 | 0.160 | 6.9x10^-03^ | 0.074 | 0.21 |
| HSPA6 | 0.462 | 1.8x10^-16^ | 0.306 | 1.3x10^-07^ |
| IFNG | 0.342 | 2.9x10^-09^ | 0.131 | 0.03 |
| KLRC3 | 0.259 | 9.4x10^-06^ | 0.208 | 4.2x10^-04^ |
| KLRC4 | 0.213 | 2.9x10^-04^ | 0.095 | 0.11 |
| KLRD1 | 0.422 | 1.0x10^-13^ | 0.245 | 2.8x10^-05^ |
| LGMN | 0.494 | 6.6x10^-19^ | 0.224 | 1.4x10^-04^ |
| NFYC TF | -0.213 | 2.9x10^-04^ | -0.212 | 3.1x10^-04^ |
| PSME1 | 0.134 | 0.02 | 0.154 | 9.3x10^-03^ |
| RFXAP TF | -0.132 | 0.03 | -0.131 | 0.03 |
| TAP1 | 0.320 | 3.4x10^-08^ | 0.210 | 3.6x10^-04^ |
| TAP2 | 0.322 | 2.7x10^-08^ | 0.222 | 1.6x10^-04^ |
| TAPBP | 0.279 | 1.7x10^-06^ | 0.231 | 8.3x10^-05^ |
| TNF | 0.164 | 5.4x10^-03^ | 0.001 | 0.99 |

**Supplemental Table 2:** **Analysis of the antigen processing and presentation pathway and gene-wise correlation with the expression of CD39 (**[ENTPD1](javascript:void(0);)) **and CD73 (NT5E).** Out of 57 genes linked to this pathway 42 genes show correlation with CD39 and 41 genes correlate with CD73.
