# Supplementary material for: Adenosine-generating ovarian cancer cells attract myeloid cells which differentiate into adenosine-generating tumor associated macrophages – a self-amplifying, CD39- and CD73-dependent mechanism for tumor immune escape
Source: J Immunother Cancer. 2016 Aug 16;4:49. doi: 10.1186/s40425-016-0154-9 (PMC4986205; doi:10.1186/s40425-016-0154-9)
Supplement: Additional file 4: Table S3. — Gene expression correlation analysis. Correlation between CD39 (ENTPD-1) or CD73 (NT5E) and phenotypic markers on human myeloid cells. Gene expression data from 285 ovarian cancer tissues from the AOCS (Australian Ovarian Cancer Study) were screened for genes correlating with the presence of CD39 (ENTPD-1) or CD73 (NT5E). Pairwise correlation analyses indicated positive correlations between almost all phenotypical markers described for MDSC (Talmadge and Gabrilovich, 2013) and the ectoenzymes CD73 and CD39. (DOCX 14 kb) [file 40425_2016_154_MOESM4_ESM.docx]

## Supplemental Table 3

|  | **CD39 ( ENTPD-1)** | | **CD73 ( NT5E)** | |
| --- | --- | --- | --- | --- |
| **HUGO** | **R** | **p** | **R** | **p** |
| AIF-1 | 0.582 | 3.1x10^-27^ | 0.404 | 1.2x10^-12^ |
| ANPEP | 0.286 | 9.2x10^-7^ | 0.259 | 9.1x10^-6^ |
| CCR2 | 0.481 | 6.9x10^-18^ | 0.320 | 3.2x10^-8^ |
| CD14 | 0.579 | 7.1x10^-27^ | 0.452 | 9.8x10^-16^ |
| CD33 | 0.488 | 1.8x10^-18^ | 0.148 | 0.01 |
| CD34 | 0.233 | 6.9x10^-5^ | 0.181 | 2.2x10^-3^ |
| CD38 | 0.337 | 5.4x10^-9^ | 0.201 | 6.6x10^-4^ |
| CD68 | 0.364 | 2.4x10^-10^ | 0.272 | 3.1x10^-6^ |
| CD80 | 0.239 | 4.6x10^-5^ | 0.042 | 0.48 |
| CD163 | 0.605 | 7.2x10^-30^ | 0.383 | 2.1x10^-11^ |
| CSF2RA | 0.426 | 5.7x10^-14^ | 0.302 | 2x10^-7^ |
| CSF2RB | 0.633 | 2.4x10^-33^ | 0.392 | 6.9x10^-12^ |
| CXCR2 | 0.207 | 4.5x10^-4^ | 0.207 | 4.3x10^-4^ |
| HLA | 0.440 | 6.3x10^-15^ | 0.407 | 8.9x10^-13^ |
| IL4R | 0.454 | 6.6x10^-16^ | 0.508 | 4.6x10^-20^ |
| ITGAM | 0.554 | 2.4x10^-24^ | 0.439 | 7.5x10^-15^ |
| ITGAX | 0.576 | 1.5x10^-26^ | 0.259 | 9.1x10^-6^ |
| KDR | 0.359 | 4.3x10^-10^ | 0.151 | 0.01 |
| LY75 | 0.055 | 0.36 | 0.288 | 7.7x10^-7^ |
| LY96 | 0.641 | 2.4x10^-34^ | 0.492 | 9.1x10^-19^ |
| MNDA | 0.604 | 1.1x10^-29^ | 0.482 | 5.6x10^-18^ |
| MS4A7 | 0.601 | 2.5x10^-29^ | 0.429 | 3.6x10^-14^ |
| PTPRC | 0.678 | 1.2x10^-39^ | 0.376 | 5.5x10^-11^ |
| TEK | 0.327 | 1.6x10^-8^ | 0.234 | 6.8x10^-5^ |

**Supplemental Table 3: Gene expression correlation analysis. Correlation between CD39 (ENTPD-1) or CD73 (NT5E) and phenotypic markers on human myeloid cells.** Gene expression data from 285 ovarian cancer tissues from the AOCS (Australian Ovarian Cancer Study) were screened for genes correlating with the presence of CD39 (ENTPD-1) or CD73 (NT5E). Pairwise correlation analyses indicated positive correlations between almost all phenotypical markers described for MDSC (Talmadge and Gabrilovich, 2013) and the ectoenzymes CD73 and CD39.
